# Temporal cytokine profiling of acute dengue, Zika, Chikungunya, and Mayaro virus infections in Northern Peru

**DOI:** 10.1186/s12879-026-12537-x

**Published:** 2026-01-17

**Authors:** Juana del Valle-Mendoza, Hugh Watson, Yordi Tarazona-Castro, Wilmer Silva-Caso, Ronald Aquino-Ortega, Hugo Carrillo-Ng, Jorge Bazan-Mayra, Victor Zavaleta-Gavidia, Miguel Angel Aguilar-Luis

**Affiliations:** 1 Biomedicine Laboratory, Research Center of The Faculty of Health Sciences, Universidad Peruana de Ciencias Aplicadas, Av. San Marcos cuadra 2, Chorrillos, Lima, Peru; 2 Quayside Research, Lyon, France; 3 Instituto de Investigacion de Enfermedades Infecciosas, Lima, Perú; 4 Regional Laboratory of Cajamarca, Regional Health Directorate (Dirección Regional de Salud, DIRESA) of Cajamarca, Cajamarca, Peru; 5 Facultad de Medicina, Universidad Nacional de Cajamarca, Cajamarca, Peru

**Keywords:** Arboviruses, Acute febrile illness, Dengue, Chikungunya, Zika, Mayaro, Cytokine, Peru

## Abstract

**Background:**

Arboviruses such as Dengue virus (DENV), Chikungunya virus (CHIKV), Zika virus (ZIKV), and Mayaro virus (MAYV) are major causes of acute febrile illness (AFI) in Peru. However, their overlapping clinical symptoms complicate differential diagnosis. Identifying immunological markers like cytokine profiles could facilitate differential diagnosis and improve understanding of their immunopathogenesis. This study aimed to describe the cytokine responses (IL-2, IL-6, IL-10, TNF-α, IFN-γ) associated with each infection.

**Methods:**

A cross-sectional study was conducted from June 2020 to April 2022 in Cajamarca, Peru, including 20 patients each with DENV, CHIKV, ZIKV, or MAYV infection, and 20 healthy controls. Infections were confirmed by RT-PCR and virus-specific IgM ELISAs. Serum cytokine levels (IL-2, IL-6, IL-10, TNF-α, IFN-γ) were measured by ELISA. Cytokine profiles were compared across the four infections and controls, and cytokine kinetics were analyzed by stratifying patients by days post-symptom onset.

**Results:**

All four infections presented with overlapping acute symptoms (e.g., fever, headache, myalgia, arthralgia). Despite these clinical similarities, each infection presented a different cytokine profile. IL-2 levels were comparable among the arbovirus groups (though higher in DENV and patients with ZIKV infection than in controls). IL-6 was elevated predominantly in CHIKV and ZIKV; IL-10 was highest in DENV; and TNF-α and IFN-γ were significantly elevated in DENV and ZIKV (with DENV levels exceeding those in MAYV). Analysis of cytokine dynamics showed that levels of all five cytokines generally peaked early (days 1–4 post-symptom onset) and declined thereafter, with the timing and magnitude of peak responses varying by virus.

**Conclusion:**

Each arboviral infection analyzed presented a distinct cytokine profile, with elevated IL-6 levels in CHIKV and ZIKV. IL-2, IL-10, TNF-α, and IFN-γ levels were elevated among the flaviviruses studied, with a delayed increase in IL-2 observed in ZIKV patients.

**Clinical trial number:**

Not applicable.

## Introduction

Acute febrile illness (AFI) represents a significant clinical challenge globally, particularly in tropical and resource-limited settings where a broad spectrum of infectious etiologies coexists [1, 2]. Among these etiologies, arboviruses constitute a critical concern due to their increasing incidence and overlapping clinical presentations [2, 3]. Pathogens such as Dengue virus (DENV) [4, 5], Chikungunya virus (CHIKV) [4–6], Zika virus (ZIKV) [4, 6, 7], and Mayaro virus (MAYV) [8] have been identified in Peru as causes of AFI.

Clinically, these arboviruses share overlapping symptoms (e.g., fever, rash, myalgia, arthralgia, and headache), making clinical differentiation difficult, particularly in regions with limited diagnostic capabilities and highlighting the need for reliable biomarkers to support accurate diagnosis and effective management [1, 9].

Recent research has highlighted cytokines as key immune mediators in arboviral pathogenesis. Cytokines orchestrate immune responses and may serve as diagnostic and prognostic biomarkers in arboviral infections [10, 11]. The lack of available vaccines is a critical consequence of our limited knowledge of interleukin-mediated immune responses in arboviral infections. Furthermore, considering the co-circulation of these pathogens in Peru, the description of cytokine profiles could contribute to facilitating diagnosis [12].

Previous research has identified distinct cytokine profiles associated with each arbovirus. DENV and CHIKV infections show distinct cytokine patterns (e.g., IL-4, IL-6, IL-8, IL-10, IL-12, IFN-α, and IFN-γ) [10, 12, 13]. Notably, IL-10 and IFN-γ were linked to dengue severity [14, 15].

In ZIKV infection, have reported elevations in cytokines such as IFN-γ, IL-1ra, IL-7, IL-9, and RANTES, associated with both mild systemic inflammation and severe neurological outcomes [16, 17].

Likewise, studies of MAYV infections have highlighted significant pro-inflammatory cytokine expressions (e.g., IL-7, IL-13, VEGF), correlated with persistent arthritic symptoms post-infection [18, 19]. Given the relatively limited literature on MAYV-induced immune responses, further investigation is needed to better define its immunopathogenesis.

Overall, arbovirus infections exhibit broad cytokine and chemokine profiles. In addition to the findings observed in DENV, increases in IL-6, IL-8, TNF-α, IL-10, chemokines such as MCP-1/CCL2 and IP-10/CXCL10, inflammasome activation (IL-1β), and, in some studies, decreased IL-12 have been described. Several markers (IL-6, IL-10, IP-10) are associated with high viremia [20]. In CHIKV, the acute phase is characterized by IFN-α/γ, IL-6, IL-8, IL-10, MCP-1, and IP-10. Meta-analyses and clinical series link IL-6 and MCP-1 with higher viral load, and specific induction of MCP-1 in monocytes has been described [21]. ZIKV exhibits a prominent chemokine bias (CCL2, CCL5, CXCL1/8/10) along with type I interferon responses; however, ZIKV can inhibit IFN-α, modulating innate immunity [22]. For MAYV, although evidence in humans is more limited, sustained pro-inflammatory profiles during convalescence and antiviral responses with induction of ISG15, IFITM1, and MX2 in macrophages and CNS cells have been reported, suggesting combined pro-inflammatory-interferon activation [23].

In Peru, the predominant vector for DENV, ZIKV, and CHIKV in urban and peri-urban areas is *Aedes aegypti*, with well-documented circulation in the Amazon region and recent expansion into coastal areas. Conversely, although *Aedes albopictus* is a competent vector in other countries, its established presence in Peru has not been reported in recent assessments [24]. Regarding MAYV, transmission is primarily sylvatic and is associated with the mosquito *Haemagogus janthinomys* in Peruvian Amazonian forests. Furthermore, vector competence studies with colonies from Iquitos show that Ae. aegypti can transmit MAYV under laboratory conditions, supporting the potential for peri-urban/urban spillover [25].

The overlapping immune responses of arboviruses call for comparative studies, especially in endemic areas like Peru, where these pathogens co-circulate frequently. To address this gap, our study aimed to describe and compare the variations in the cytokines IL-2, IL-6, IL-10, TNF-α, and IFN-γ in DENV, CHIKV, ZIKV, and MAYV infections in a northeastern region of Peru. By describing and characterizing the cytokine profile, this research seeks to contribute to diagnosis and improve clinical outcomes in regions disproportionately affected by arboviral diseases.

## Materials and methods

### Study location and design

This cross-sectional study was conducted in the department of Cajamarca, located in northeastern Peru, between June 2020 and April 2022. Cajamarca is located at approximately 2,750 m above sea level (9,022 feet) and has a population of approximately 1,341,012 inhabitants. The region has previously reported diverse etiological agents responsible for acute febrile illness (AFI), including DENV, CHIKV, ZIKV, MAYV, as well as bacterial pathogens such as *Leptospira* spp., *Bartonella* spp., *Rickettsia* spp [26, 27]. Five distinct patient groups (DENV, CHIKV, ZIKV, MAYV, and healthy controls) comprising 20 patients each were analyzed to compare cytokine profiles.

### Case definition and sample collection

Patients were enrolled through Peru’s ongoing national syndromic surveillance program for acute febrile illnesses from primary healthcare centers. Eligible patients presented with AFI defined as body temperature above 38 °C and non-specific symptoms such as arthralgia, headache, myalgia, among others, without an identifiable source of infection (e.g., pneumonia or urinary tract infection). Inclusion required laboratory confirmation of arboviral infection by RT-PCR and positive IgM serology specific for DENV, CHIKV, ZIKV, or MAYV. Healthy controls (HC) included febrile patients negative for these arboviruses. Exclusion criteria included autoimmune diseases, immunodeficiency, immunosuppressive drug use, and pregnancy.

Blood samples were collected using the Vacuette TUBE Serum Separator Clot Activator (Greiner Bio-One, Kremsmünster, Austria). Samples were immediately centrifuged, and sera were stored at − 80 °C until transported to Lima, Peru, for further laboratory analysis.

### Detection of DENV, CHIKV, ZIKV, and MAYV

Specific arbovirus infections were diagnosed initially by clinical suspicion and subsequently confirmed using RT-PCR described by Alva-Urcia et al. [4], Palomares-Reyes [5] and Sánchez-Carbonel [6].

### Cytokine analysis

Cytokine levels (IL-2, IL-6, IL-10, TNF-α, and IFN-γ) were measured in duplicate using High-Sensitivity Human ELISA Kits (Abcam, MA, United States): IL-2 (Ref. ab46054), IL-6 (Ref. ab46027), IL-10 (Ref. ab46059), TNF-α (Ref. ab181421), and IFN-γ (Ref. ab46048), following the manufacturer’s protocol. Cytokine concentrations were reported in pg/mL.

### Statistical analysis

Demographic data, clinical features, and cytokine concentrations were compiled into a structured dataset. Descriptive statistics summarized demographic and clinical characteristics, expressed as frequencies (percentages) for categorical variables and medians with interquartile ranges (IQR) for continuous variables.

Statistical analyses were performed using Stata 18.0 (StataCorp LLC, College Station, TX, USA) and OriginPro 2025 (OriginLab Corporation, Northampton, MA, USA). Normality was assessed with the Shapiro-Wilk test. Non-normally distributed data were analyzed with the Kruskal-Wallis test followed by Dunn’s post-hoc test for multiple comparisons between groups. Additionally, the p-values obtained from Dunn’s test were adjusted using the Bonferroni correction method. Correlation analyses between cytokine levels and demographic variables (gender, age, and days post symptom onset) were performed using Spearman’s rank correlation coefficient. Hierarchical clustering analysis utilized Z-score standardized cytokine values, employing Euclidean distance and the average linkage method. Results were visualized using boxplots and heatmaps generated in OriginPro 2025. Statistical significance was set at *p* < 0.05.

### Ethics statement

The study protocol was approved by the Research Ethics Board of the Direccion Regional de Salud de Cajamarca, Peru (Nº document: MAD 08547925). All samples were collected within the epidemiological/syndromic surveillance framework according to the guidelines of Peru’s Ministry of Health National Center for Epidemiology, Disease Control, and Prevention. All participants gave their consent to participate in the study, informed consent was obtained from all participants and the study procedures adhered to the Declaration of Helsinki. All procedures were performed under the international ethics guidelines for research in human healthcare issued by the Council for International Organizations of Medical Sciences (CIOMS) and the World Health Organization (WHO).

## Results

### Demographic and clinical characteristics

Demographic and clinical characteristics of participants are summarized in Table 1. The study included 100 participants, comprising 20 patients each infected with DENV, CHIKV, ZIKV, MAYV, and 20 healthy controls (HC). Age (χ² (8) = 12.03, *p* = 0.151) and gender (χ²(4) = 3.52, *p* = 0.514) distributions were similar across groups.

**Table 1:** Demographic characteristics, days of fever, clinical manifestations in patients infected with DENV, CHIKV, ZIKV, MAYV, and healthy control

	DENV *n* = 20 (%)	CHIKV *n* = 20 (%)	ZIKV *n* = 20 (%)	MAYV *n* = 20 (%)	Controls *n* = 20 (%)	*P*-value
**Age groups (years)**						0.151
≤ 17	1 (5.0)	4 (20.0)	1 (5.0)	4 (20.0)	1 (5.0)	
18–39	11 (55.0)	10 (50.0)	14 (70.0)	5 (25.0)	10 (50.0)	
≥ 40	8 (40.0)	6 (30.0)	5 (25.0)	11 (55.0)	9 (45.0)	
**Gender**						0.514
Male	10 (50.0)	6 (30.0)	5 (25.0)	8 (40.0)	6 (30.0)	
Female	10 (50.0)	14 (70.0)	15 (75.0)	12 (60.0)	14 (70.0)	
**Days post symptom onset (DPSO)**						**0.001**
1–2 days	5 (25.0)	7 (35.0)	7 (35.0)	6 (30.0)		
3–4 days	12 (60.0)	7 (35.0)	9 (45.0)	8 (40.0)		
5–9 days	3 (15.0)	6 (30.0)	4 (20.0)	6 (30.0)		
**Clinical symptoms**						
Arthralgias	15 (75.0)	14 (70.0)	11 (55.0)	10 (50.0)	13 (65.0)	0.492
Polyarthralgias in hands	10 (50.0)	4 (20.0)	7 (35.0)	9 (45.0)	7 (35.0)	0.330
Polyarthralgias in feet	10 (50.0)	4 (20.0)	4 (20.0)	8 (40.0)	4 (20.0)	0.076
Arthritis	1 (05.0)	0 (00.0)	4 (20.0)	2 (10.0)	4 (20.0)	0.162
Myalgias	14 (70.0)	13 (65.0)	11 (55.0)	10 (50.0)	12 (60.0)	0.774
Headache	19 (95.0)	14 (70.0)	15 (75.0)	18 (90.0)	16 (80.0)	0.227
Ocular/retroocular pain	7 (35.0)	10 (50.0)	10 (50.0)	8 (40.0)	10 (50.0)	0.804
Lumbar pain	6 (30.0)	3 (15.0)	10 (50.0)	6 (30.0)	8 (40.0)	0.198
Rash	3 (15.0)	1 (05.0)	5 (25.0)	2 (10.0)	1 (05.0)	0.379
Conjunctivitis	-	-	1 (05.0)	-	3 (15.0)	0.122
Nausea	6 (30.0)	7 (35.0)	6 (30.0)	11 (55.0)	6 (30.0)	0.454
Vomiting	6 (30.0)	7 (35.0)	4 (20.0)	10 (50.0)	2 (10.0)	0.064
**Warning signs**						
Persistent vomiting				1 (5.0)		1.000
Severe and persistent abdominal pain	1 (05.0)	2 (10.0)	0 (00.0)	2 (10.0)		0.554
Hepatomegaly	1 (5)					1.000

Values are presented as absolute numbers with percentages in parentheses for categorical variables. Statistical comparisons between groups were performed Chi-square/Fisher’s exact test for categorical variables. DENV: Dengue virus; CHIKV: Chikungunya virus; ZIKV: Zika virus; MAYV: Mayaro virus. Bold values indicate statistical significance (*p* < 0.05)

Clinical manifestations such as arthralgia, polyarthralgia in hands and feet, myalgia, headache, ocular pain, lumbar pain, rash, nausea, conjunctivitis and vomiting were prevalent among patients with no statistically significant differences between groups (all p-value > 0.05). Severe symptoms like persistent vomiting, severe abdominal pain, and hepatomegaly were infrequently observed (Table 1).

Days post symptom onset (DPSO) were classified into three intervals (1–2, 3–4, 5–9 days). A significant difference in DPSO distribution among infection groups was detected (χ² (12) = 104.27, *p* < 0.001).

### Cytokine expression profiles

**Fig. 1:**
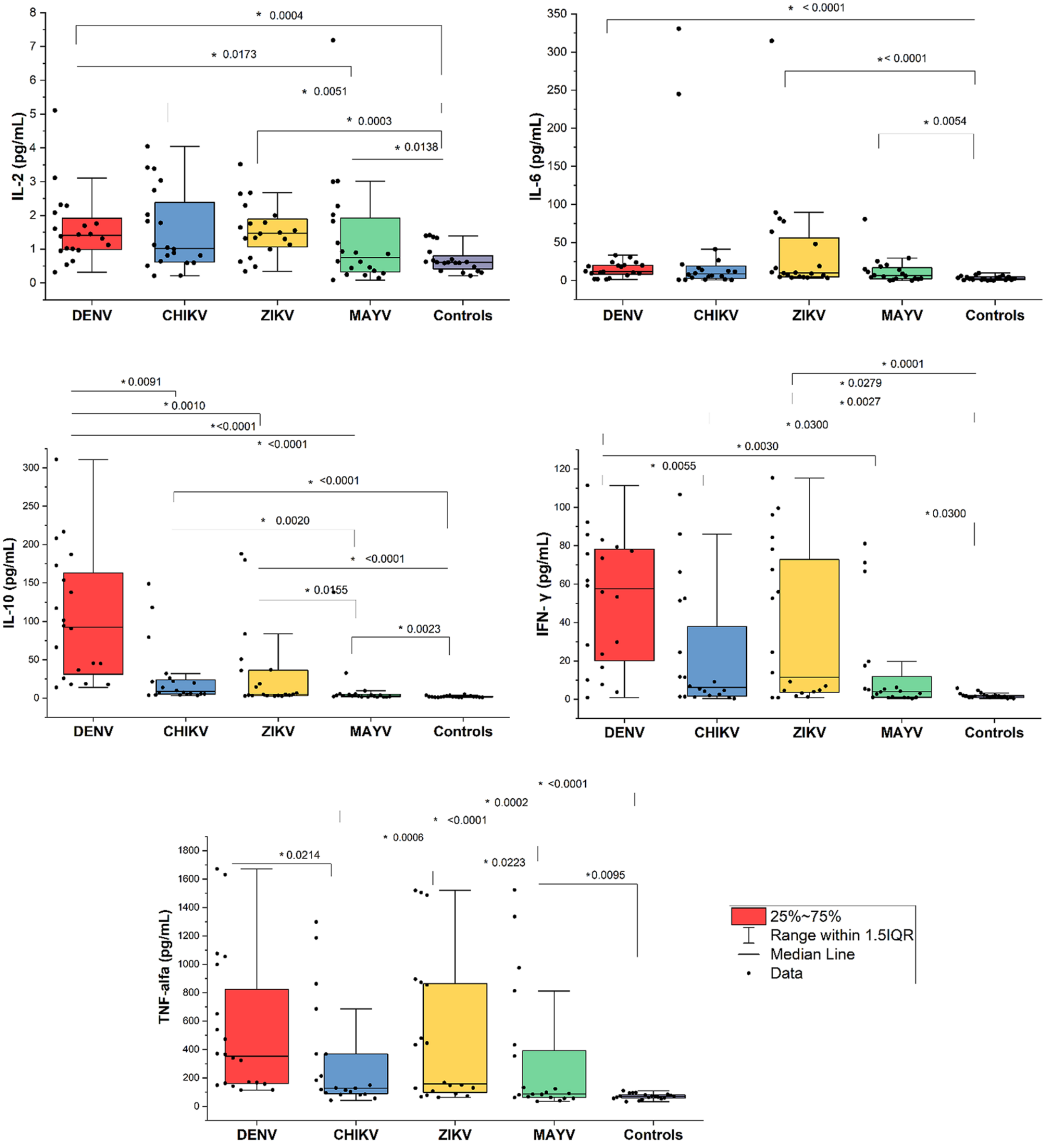
Comparative analysis of cytokine levels in patients with arboviral infections and controls. Boxplots illustrate serum concentrations of IL-2, IL-6, IL-10, TNF-α, and IFN-γ across patients infected with Dengue virus (DENV), Chikungunya virus (CHIKV), Zika virus (ZIKV), Mayaro virus (MAYV), and healthy controls (HC). Cytokine levels were measured in pg/mL using high-sensitivity ELISA assays. The boxes represent the interquartile range (IQR), the horizontal line indicates the median, and whiskers denote the range within 1.5 times the IQR. Individual data points are shown as black dots. Statistical comparisons between groups were performed using the Kruskal-Wallis test followed by Dunn’s post hoc test. Significant *p-values* (< 0.05) are indicated above the corresponding comparisons, highlighting differential cytokine expression patterns among arboviral infections and controls

Given the non-normal distribution of cytokine levels confirmed by the Shapiro-Wilk test (*p* < 0.05), data are presented as median and interquartile range (IQR). Significant differences across groups were identified for all cytokines assessed (IL-2, IL-6, IL-10, TNF-α, and IFN-γ; all p-value < 0.001, Kruskal-Wallis test; Table 2; Fig. 1). Post-hoc Dunn’s tests revealed specific patterns:

**Table 2:** Serum cytokine levels (pg/mL) in patients with DENV, CHIKV, ZIKV, MAYV infections, and healthy controls

Cytokine level (pg/mL)	DENV *n* = 20 (%)	CHIKV *n* = 20 (%)	ZIKV *n* = 20 (%)	MAYV *n* = 20 (%)	Controls *n* = 20 (%)	*P*-value
IL-2	1.4 (1–2)	1.0 (1–2)	1.5 (1–2)	0.8 (0–2)	0.6 (0–1)	**0.0016**
IL-6	11.9 (8–20)	8.7 (3–19)	10.1 (5–56)	6.5 (2–17)	2.3 (1–5)	**0.0001**
IL-10	92.4 (31–163)	8.6 (5–24)	4.8 (3–36)	3.0 (2–5)	1.6 (1–2)	**0.0001**
TNF-α	352.4 (160–824)	128.7 (90–368)	158.1 (98–864)	87.7 (62–393)	69.4 (58–81)	**0.0001**
IFN-γ	57.6 (20–78)	6.1 (2–38)	11.5 (4–73)	4.0 (1–12)	1.6 (1–2)	**0.0001**

Cytokine concentrations are expressed as median (interquartile range, IQR). Statistical comparisons were performed using the Kruskal–Wallis test followed by Dunn’s post hoc test with Bonferroni correction. DENV: Dengue virus; CHIKV: Chikungunya virus; ZIKV: Zika virus; MAYV: Mayaro virus. Bold values indicate statistical significance (*p* < 0.05)

- IL-2 levels were significantly elevated in DENV (*p* = 0.0004) and ZIKV (*p* = 0.0003) patients compared to healthy controls (HC). Additionally, IL-2 was significantly higher in DENV compared to MAYV (*p* = 0.0173).
- IL-6 concentrations were significantly higher in DENV (*p* < 0.0001), CHIKV (*p* = 0.0008), and ZIKV (*p* < 0.0001) compared to controls. However, no significant differences were noted among arboviral infection groups.
- IL-10 was significantly elevated in DENV patients compared to CHIKV (*p* = 0.0091), ZIKV (*p* = 0.0010), MAYV (*p* < 0.0001), and controls (*p* < 0.0001). IL-10 was also significantly higher in CHIKV and ZIKV compared to MAYV and controls (*p* < 0.05).
- TNF-α concentrations were significantly increased in DENV patients compared to CHIKV (*p* = 0.0214), MAYV (*p* = 0.0006), and controls (*p* < 0.0001). Moreover, ZIKV and CHIKV groups exhibited significantly higher TNF-α levels compared to controls (*p* < 0.0002).
- IFN-γ levels were significantly elevated in DENV compared to CHIKV (*p* = 0.0055), MAYV (*p* = 0.0003), and controls (*p* < 0.0001). IFN-γ was also significantly higher in ZIKV and CHIKV patients compared to controls (*p* < 0.003).

These findings demonstrate distinctive cytokine expression profiles across arboviral infections, emphasizing significant inflammatory and regulatory cytokine responses particularly pronounced in DENV infection.

### Hierarchical clustering of cytokine profiles

**Fig. 2:**
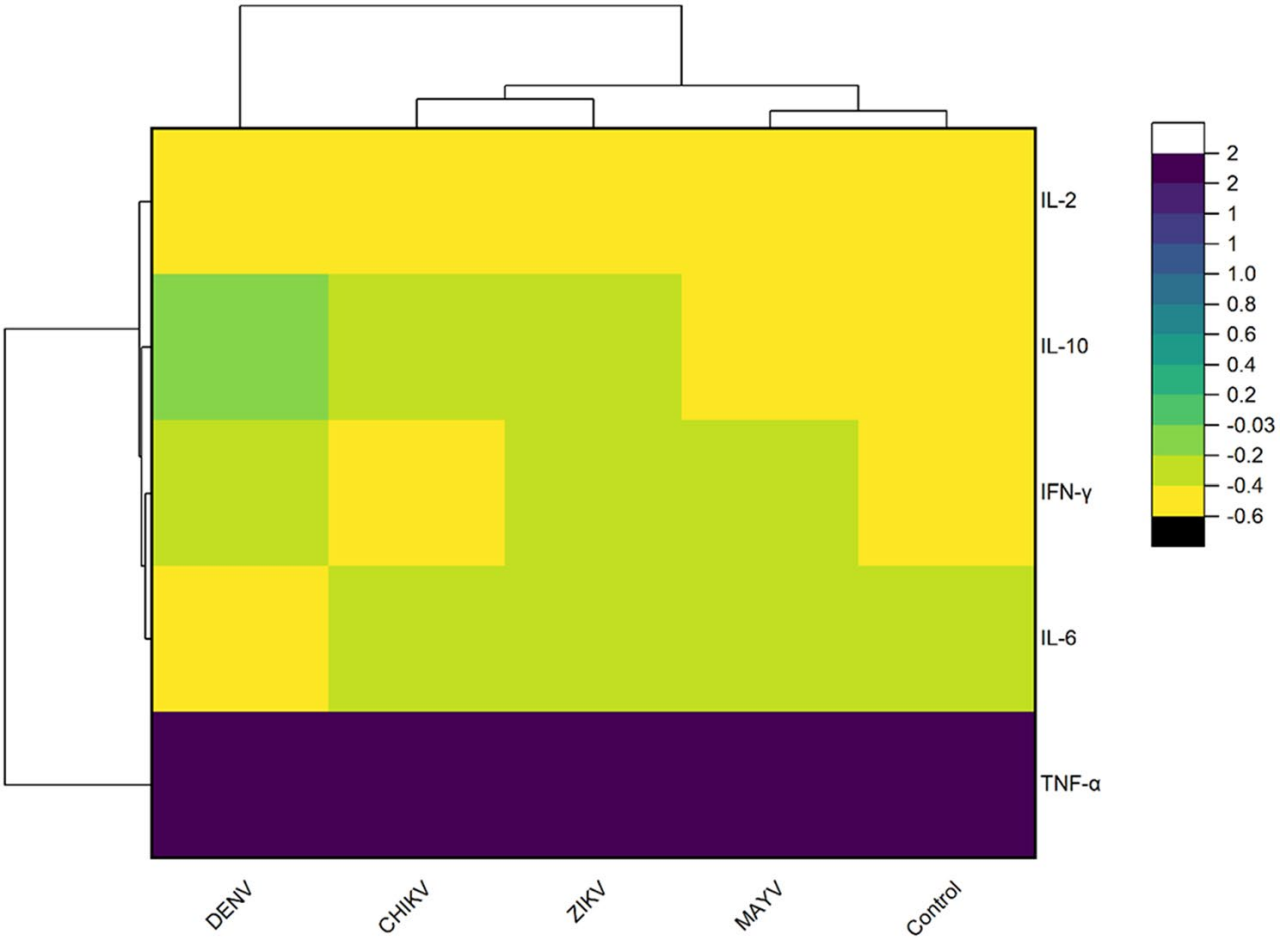
Hierarchical clustering heatmap of standardized cytokine expression across patients with arboviral infections and controls. Z-score standardized values of IL-2, IL-6, IL-10, TNF-α, and IFN-γ were compared among patients infected with Dengue virus (DENV), Chikungunya virus (CHIKV), Zika virus (ZIKV), Mayaro virus (MAYV), and healthy controls. Hierarchical clustering was applied to both cytokines and patient groups using Euclidean distance and the average linkage method. The color scale represents standardized cytokine expression levels, where purple indicates higher expression and yellow indicates lower expression relative to the mean of each cytokine

Hierarchical clustering highlighted distinct cytokine expression patterns among arboviral infections and healthy controls (HC) (Fig. 2). Cytokines are clustered into two major groups: pro-inflammatory cytokines (TNF-α, IFN-γ, IL-6) and immunomodulatory cytokines (IL-2, IL-10). Patients with DENV displayed a unique cytokine profile characterized by elevated IL-10, TNF-α, and IFN-γ.

### Correlation of cytokine levels with patient variables

**Fig. 3:**
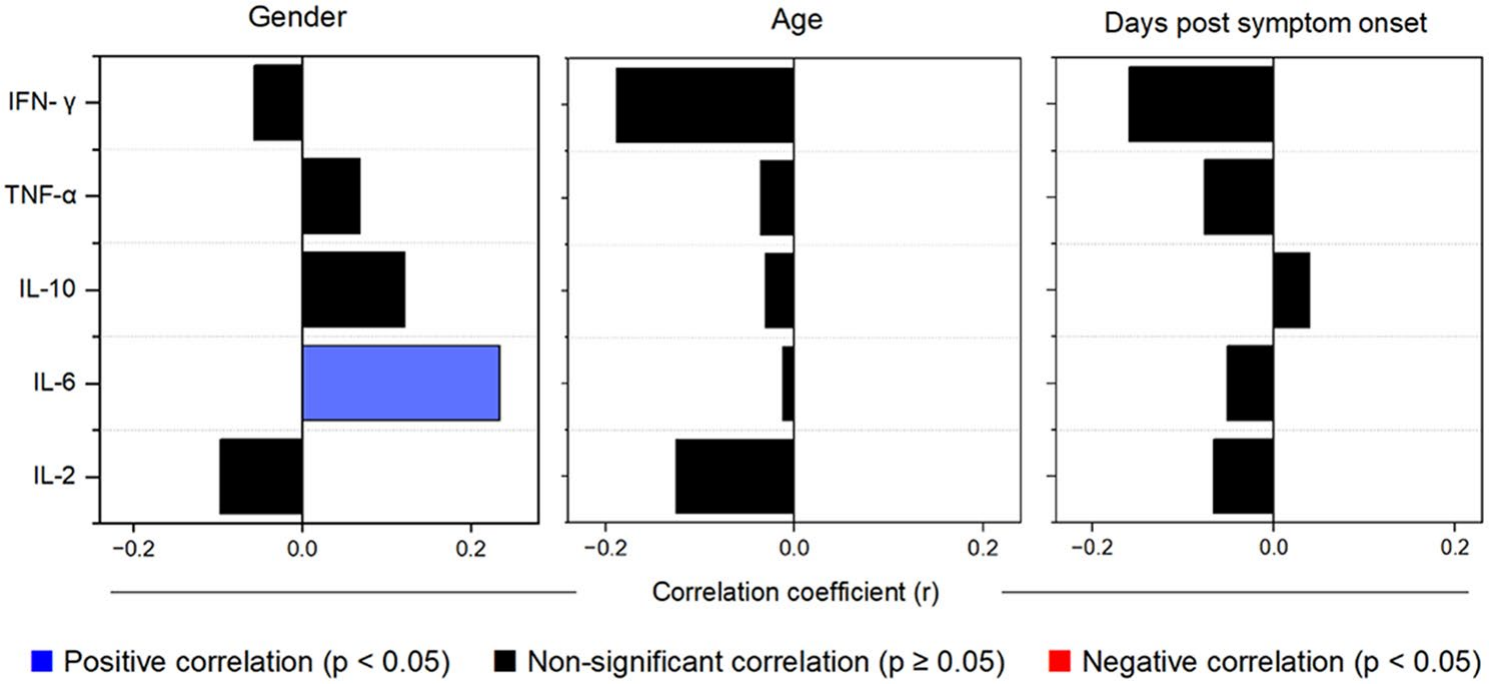
Correlation analysis between cytokine levels and patient variables: gender, age, and days post symptom onset. Spearman’s correlation coefficients (r) between serum cytokine concentrations (IL-2, IL-6, IL-10, TNF-α, IFN-γ) and patient characteristics. Significant positive correlations (p-value < 0.05) are shown in blue, while non-significant correlations are displayed in black. No significant negative correlations were observed

Spearman’s correlation analyses did not reveal significant correlations between cytokine levels and Days Post Symptom Onset (DPSO) or age (all *p* > 0.05, Fig. 3). However, IL-6 exhibited a weak but significant positive correlation with gender (*r* = 0.2342, *p* = 0.019), suggesting potential gender-related differences in IL-6 expression.

Despite the lack of significant correlations, further exploratory analyses were conducted by stratifying samples into specific DPSO intervals (days 1–2, 3–4, and 5–9) to compare cytokine kinetics among infected patients and healthy controls, providing additional insights into cytokine expression dynamics during infection progression.

### Temporal dynamics of cytokine levels

**Fig. 4:**
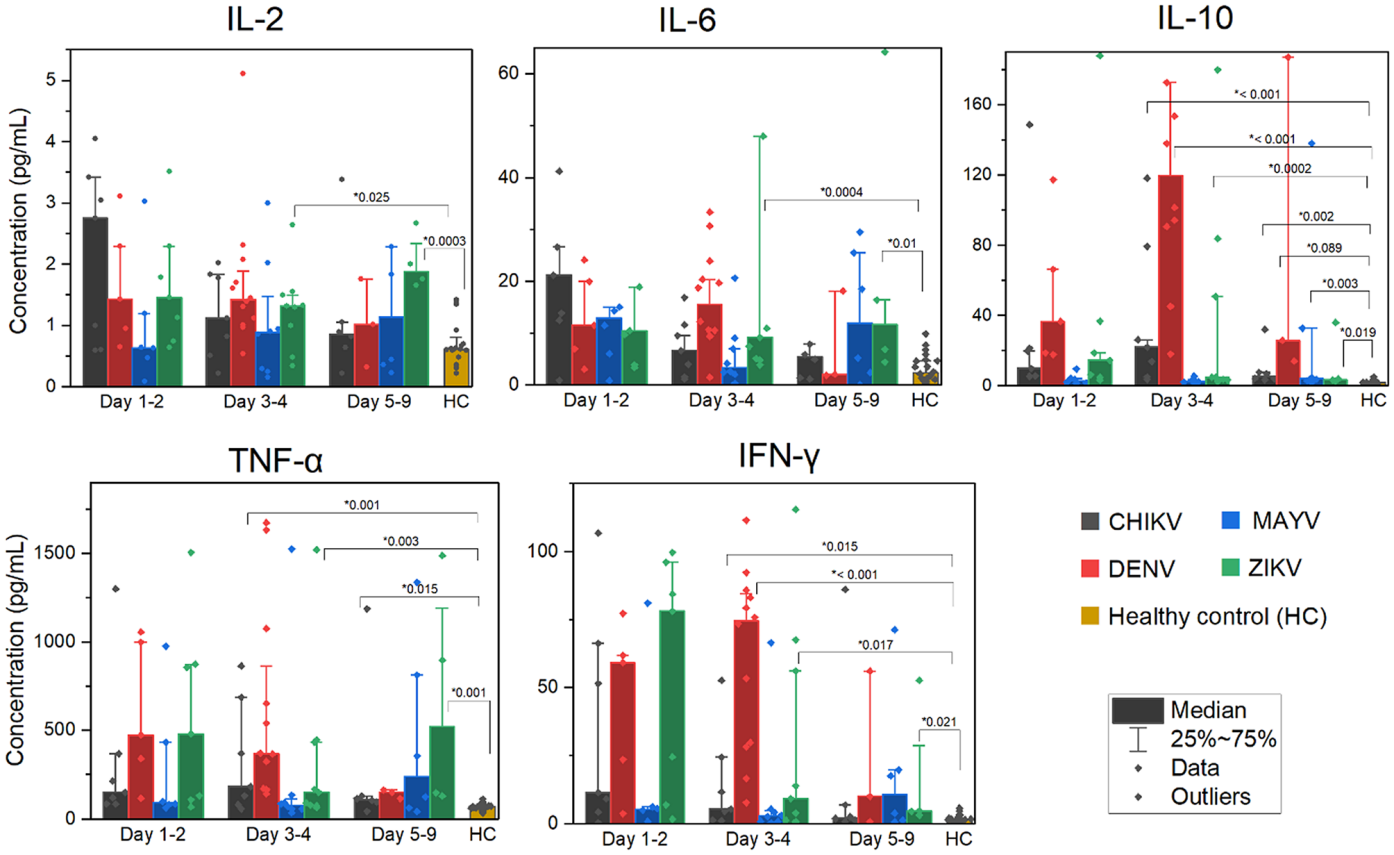
Temporal dynamics of serum cytokine levels in patients with arboviral infections according to days post symptom onset. Median concentrations and interquartile ranges (IQR) of IL-2, IL-6, IL-10, TNF-α, and IFN-γ in patients infected with DENV, CHIKV, ZIKV, and MAYV, stratified by days post symptom onset (1–2, 3–4, and 5–9 days) compared to healthy controls (HC). Statistical comparisons were performed using the Kruskal-Wallis test followed by Dunn’s post hoc test (adjusted Bonferroni method). Only statistically significant p-values (*p* < 0.05) are shown for days 3–4 and 5–9. All comparisons for day 1–2 versus controls were significant across cytokines and viruses, except for MAYV, where IL-2 and IFN-γ levels did not differ significantly from controls

The temporal dynamics of cytokine responses stratified by days post symptom onset (DPSO) were analyzed, revealing distinct cytokine trajectories for each arboviral infection (Fig. 4).

In DENV infection, significant increases were evident across all cytokines during days 1–2 and 3–4 compared to healthy controls (HC) (*p* ≤ 0.0054). Notably, IL-10 and IFN-γ peaked significantly at days 3–4 DPSO (*p* < 0.001). Generally, cytokine levels decreased toward days 5–9.

In CHIKV-infected patients, early elevations were prominent for TNF-α, IL-6, IL-10, and IFN-γ at days 1–2 DPSO compared to HC (*p* ≤ 0.0023). IL-10 levels remained elevated during the 3–4 DPSO interval (*p* < 0.0001), with significant decreases noted by 5–9 days.

ZIKV infection exhibited pronounced early-phase cytokine responses with significantly elevated TNF-α, IL-6, IL-10, IL-2, and IFN-γ at both days 1–2 and 3–4 compared to HC (*p* ≤ 0.0034). Importantly, a significant increase in IL-2 levels was observed during days 5–9 relative to days 3–4 (*p* = 0.0363), highlighting a progressive upregulation over the course of infection.

MAYV infection exhibited modest cytokine increases during the early phase (days 1–2 DPSO), with statistically significant differences in TNF-α, IL-6, and IL-10 levels compared to healthy controls (*p* ≤ 0.0271). In contrast, cytokine fluctuations in MAYV were less pronounced over time, showing limited variation compared to other viral infections.

Overall, these analyses underscore specific temporal dynamics, where cytokine levels generally peak early in the disease course and subsequently decline, with particular distinctions evident among different arboviruses.

## Discussion

This study presents a comparative evaluation of cytokine levels of dengue virus (DENV), chikungunya virus (CHIKV), Zika virus (ZIKV), and Mayaro virus (MAYV) in a northeastern region of Peru. Although these arboviruses share a similar ecological and epidemiological context, the description and comparison of their cytokine responses remains poorly understood. Consistent with existing literature, our clinical data confirm that these infections often produce overlapping acute manifestations, such as fever, headache, myalgia, and arthralgia, which complicates clinical diagnosis based on symptoms alone [3, 28]. The observed variations in cytokine expression across viruses, particularly in IFN-γ, IL-10, and IL-2, reveal distinct immune activation patterns that may reflect virus-specific host responses.

DENV infection induced significant elevations in all measured cytokines during the acute phase. IL-6 and IL-10 levels were particularly high in DENV patients, in line with previous studies linking these cytokines to severe dengue disease and immune dysregulation [15, 29, 30]. Notably, DENV elicited the highest IL-10 concentrations among the four infections. Elevated IL-10 has been associated with worse outcomes in dengue and can mediate immune suppression via T-cell apoptosis [14, 31]. Robust TNF-α and IFN-γ responses were also observed in DENV patients, cytokines known to contribute to vascular leakage and severe manifestations of dengue fever [15, 32]. These findings reaffirm that dengue triggers an aggressive early immune response, which may underlie its potential for severe clinical complications.

CHIKV infection also provoked a cytokine response, with some differences from DENV. In our CHIKV patients, IL-6, IL-10, TNF-α, and IFN-γ levels were elevated relative to healthy controls, whereas IL-2 showed no notable increase. The prominence of IL-6 in acute CHIKV infection is consistent with reports associating high IL-6 with greater disease severity and prolonged arthralgia in Chikungunya [33, 34]. CHIKV also induced an early surge of IL-10 [19], although IL-10 levels in CHIKV were lower than those observed in DENV patients. As expected, TNF-α was elevated in CHIKV infection, reflecting this virus’s arthritogenic nature [35]; however, the TNF-α response in CHIKV was not significantly higher than that in DENV or ZIKV. This suggests that dengue and Zika viruses can elicit inflammatory responses comparable to CHIKV’s, even though CHIKV is classically associated with more prominent arthritis and inflammation.

We found significant elevations of IL-2, IL-6, IL-10, TNF-α, and IFN-γ in patients with ZIKV infection compared to controls. In particular, IL-6 was markedly elevated, consistent with reports of substantial systemic inflammation in acute Zika [36]. ZIKV also induced TNF-α and IFN-γ responses; although ZIKV illness is often clinically milder than dengue, such proinflammatory mediators may contribute to Zika’s neuropathogenic complications [37]. An interesting distinction was observed in the timing of responses: IL-2 levels in ZIKV cases rose later in the course of infection (peaking around days 5–9 post-onset) compared to the other viruses, indicating a somewhat delayed T-cell activation phase. Overall, our results suggest that ZIKV can trigger an acute immune response nearly as intense as that of DENV, despite generally causing less severe acute disease.

In contrast, MAYV infection exhibited a more subdued cytokine profile. MAYV patients showed only modest early increases in inflammatory cytokines compared to controls, and these increases were generally lower than those observed with the other arboviruses. Notably, IL-6 was not significantly elevated in our acute MAYV cases, contrasting with a previous report that documented IL-6 increases in MAYV infection [18, 19]. Among the four viruses, MAYV induced the lowest IL-10 levels, and its TNF-α and IFN-γ responses were also comparatively mild. Indeed, TNF-α levels were significantly lower in MAYV patients than in DENV patients, despite the expectation that an alphavirus would elicit high TNF-α production [3]. This finding suggests that acute MAYV triggers a milder immediate immune response, though sustained pro-inflammatory cytokine production has been noted in MAYV infections with prolonged arthralgia [18, 38].

Hierarchical clustering analysis further supported the distinct immunological profiles of each infection. DENV samples formed a unique cluster, predominantly driven by IL-10 and IFN-γ, aligning with their known roles in immune suppression and vascular permeability, both hallmarks of severe dengue [15, 29]. ZIKV and CHIKV showed overlapping clustering, associated with high IL-6 and TNF-α, reflecting robust pro-inflammatory responses implicated in joint and neurological complications [13, 17]. In contrast, MAYV presented a diffuse clustering pattern, consistent with its milder cytokine activation [39]. These patterns, underscore the potential of cytokine profiles to differentiate arbovirus infections that present similar clinical features.

It has been reported that each arboviral infection exhibits distinct temporal dynamics in the cytokine profile produced [12]. DENV (a flavivirus) triggers an early, marked rise in multiple cytokines; IL-10 and IFN-γ reach particularly high levels around days 3–4 after symptom onset [15], consistent with the role of IL-10 as a severity-associated factor in dengue with warning signs or severe dengue [40]. Similarly, Zika elicits a systemic inflammatory response during the acute phase: patients at days 1–4 show significant elevations of TNF-α, IL-6, IL-10, IL-2, and IFN-γ compared with healthy controls which contrasts with our results [41]. Moreover, IL-2 has been reported to continue increasing into the second week of ZIKV infection, suggesting sustained T-cell activation as the illness progresses [42, 43].

By contrast, CHIKV is characterized by a robust, immediate innate response: during days 1–2 after symptom onset, very high levels of TNF-α, IL-6, IL-10, and IFN-γ are detected relative to healthy individuals [44]. In CHIKV, IL-10 remains elevated during the first days (modulating excessive inflammation) but tends to decline by the second week as the acute febrile phase abates [42]. In MAYV infection (alphavirus), TNF-α, IL-6, and IL-10 have been documented during the first days; however, the magnitude of the response is lower than in DENV, CHIKV, or ZIKV [45, 46], as also shown in our study. In addition, cytokine fluctuations over time in MAYV appear less pronounced, consistent with clinical observations that Mayaro generally causes moderate acute illness. Nevertheless, even with this attenuated profile, MAYV can induce sufficient inflammation to contribute to prolonged arthralgias in some patients (associated with persistently elevated proinflammatory cytokines) [46].

Taken together, these temporal patterns highlight inter-arbovirus differences in cytokine levels: all trigger an early cytokine peak, but the intensity and duration of this response vary by virus, potentially shaping immunopathogenesis and the characteristic severity of each disease. This pattern aligns with prior studies showing IFN-γ as an early Th1-related cytokine and IL-10 as a later immunoregulatory mediator during the host response to DENV, ZIKV, and CHIKV infections [42].

Of particular note was the delayed increase in IL-2 observed in ZIKV-infected patients (days 5–9 DPSO), which was not equally prominent in other arboviral groups. This delayed expression may reflect a slower adaptive T-cell–mediated immune activation. Similar kinetics have been reported in ZIKV-infected nonhuman primates, where IL-2 levels increase significantly alongside CD4 + and CD8 + T-cell expansion between days 6 and 9 post-infection, supporting the notion of a distinct late-phase immune signature for ZIKV [36, 43]. These cytokine kinetic differences may have diagnostic or monitoring implications and warrant further investigation in larger longitudinal cohorts.

Our findings delineate a virus-specific inflammatory signature. DENV displayed the most pronounced profile, with IL-2, TNF-α, IFN-γ, and IL-10 exceeding healthy controls and, in several comparisons, surpassing other viruses; this aligns with literature linking DENV to peaks of IL-6, TNF-α, IL-10, MCP-1, and CXCL10, and even inflammasome activation [20]. IL-6 was elevated in DENV, CHIKV, and ZIKV versus controls, with no between-infection differences, suggesting a shared early pro-inflammatory axis [20–22]. IL-10 was higher in DENV than in CHIKV, ZIKV, and MAYV, and CHIKV/ZIKV exceeded MAYV and controls, consistent with immunoregulatory responses described in DENV and the mixed profile reported for CHIKV [20, 21]. IFN-γ increased in DENV (vs. CHIKV, MAYV, and controls) and also in ZIKV/CHIKV vs. controls, in line with interferon-pathway induction reported for ZIKV and CHIKV [21, 22]. By contrast, MAYV showed relatively lower IL-2/IL-10, concordant with sustained pro-inflammatory patterns and ISG induction rather than regulatory peaks [23]. Taken together, the overlap (e.g., IL-6, IL-10) and the distinctive signals (dominant TNF-α/IFN-γ/IL-2 in DENV) contextualize our data and underscore the importance of comparing cytokine magnitude and directionality across infections [20–23].

Although the cytokines evaluated in this study are classical immune mediators, our work provides original value by comparatively characterizing the immune response in DENV, ZIKV, CHIKV, and MAYV infections within the same cohort, using a standardized methodology and stratification by days post symptom onset in the study region. Previous studies have compared cytokine profiles between CHIKV and DENV in tropical areas such as Southeast Asia, identifying distinctive expression patterns in IL-4, IL-8, IL-13, and MCP-3, among others, thereby supporting the relevance of our comparative approach [45]. Moreover, the immunological characterization of MAYV remains limited, with few studies describing cytokine dynamics in human infections [46]. Our findings contribute novel data on the kinetics and regional variability of immune responses in arboviral infections, particularly among underrepresented Latin American populations.

### Limitations

Despite the valuable insights gained from our study, it is essential to acknowledge certain limitations. First, we collected only a single blood sample per patient, which prevented analysis of the dynamic changes in cytokine levels throughout the course of infection. Second, the panel of cytokines we measured was limited to five key mediators, and other important immune factors were not assessed; future studies should include a broader array of cytokines to capture a more complete picture of the immune response. Third, our sample size was relatively modest (20 patients per group) and drawn from a single geographic region, which may limit the generalizability of the findings. Other limitation of this study is the lack of stratification by clinical severity, due to the absence of a standardized classification for ZIKV, CHIKV, and MAYV, and the low frequency of warning signs in our cohort. This heterogeneity may have influenced the observed cytokine levels. Future studies with larger sample sizes and longitudinal designs will help adjust for clinical severity and validate the trends reported.

## Conclusion

A differential cytokine profile was described in each arboviral infection analyzed. Levels of IL-2, IL-10, TNF-α, and IFN-γ were elevated in the studied flaviviruses, with a delayed increase in IL-2 observed in patients with ZIKV. Differences in cytokine profile could serve as potential biomarkers to differentiate clinically overlapping infections and guide targeted therapies. Overall, our comparative findings provide a valuable basis for future research aimed at unraveling the mechanisms underlying these immune responses and developing better diagnostic and therapeutic strategies for arboviral diseases.

## Data Availability

Abstraction format used in the study and dataset are available and accessible at figshare in the link: 10.6084/m9.figshare.23972205.

## Abbreviations

AFI: Acute febrile illness
CHIKV: Chikungunya virus
ZIKV: Zika virus
MAY: Mayaro virus
RT-PCR: Reverse Transcription Polymerase Chain Reaction
IL-2: Interleukin-2
IL-6: Interleukin-6
IL-10: Interleukin-10, TNF-α:Tumor Necrosis Factor-alpha
IFN-γ: Interferon gamma
